# Is the Triage System Welcomed in the Tertiary Hospital of the Limpopo Province? A Qualitative Study on Patient’s Perceptions

**DOI:** 10.3390/nursrep13010033

**Published:** 2023-02-27

**Authors:** Thabo Arthur Phukubye, Tshepo Albert Ntho, Livhuwani Muthelo, Masenyani Oupa Mbombi, Mamare Adelaide Bopape, Tebogo Maria Mothiba

**Affiliations:** 1 Department of Nursing, University of Limpopo, Private Bag X1106, Polokwane 0727, South Africa; 2 Faculty of Health Science Executive Dean’s Office, University of Limpopo, Private Bag X1106, Polokwane 0727, South Africa

**Keywords:** triage system, patients, perception, tertiary hospital, qualitative research

## Abstract

A triage system in the emergency department is necessary to prioritize and allocate scarce health resources to the medical needs of the patients to facilitate quality health service delivery. This paper aimed to ascertain if the triage system is welcomed in the tertiary hospital of Limpopo Province by exploring patients’ perceptions in the emergency department in South Africa. A qualitative research approach was used in this study with descriptive, explorative, and contextual research design to reach the research objective. Purposive sampling was used to select the patients who participated in semi-structured one-on-one interviews, which lasted between 30 and 45 min. The sample size was determined by data saturation after 14 participants were interviewed. A narrative qualitative analysis method was used to interpret and categorize the patients’ perceptions into seven domains of Benner’s theory. The six relevant domains illustrated mixed patients‘ perceptions regarding the triage system in the emergency departments. The domain-helping role of the triage system was overweighed by the dissatisfaction of the needy patients who waited for an extended period to receive emergency services. We conclude that the triage system at the selected tertiary hospital is not welcomed due to its disorganization and patient-related factors in the emergency departments. The findings of this paper are a point of reference for reinforcing the triage practice and improved quality service delivery by the emergency department healthcare professionals and the department of health policymakers. Furthermore, the authors propose that the seven domains of Benner’s theory can serve as a foundation for research and improving triage practice within emergency departments.

## 1. Introduction

Emergency departments are the most challenging units worldwide due to daily overcrowding that impacts health service delivery [1]. Overcrowding of the emergency department or unit is defined as the situation in which emergency department function is impeded primarily because of the excessive number of patients waiting to be seen, under-going assessment and treatment, or waiting for departure compared to the physical or staffing capacity of the emergency department [2]. Overcrowding in the emergency department is a global problem associated with various outcomes such as staff burnout, prolonged waiting time, high patient mortality from reduced correct, timely, and efficient hospital care, and altered triage [3,4]. A triage system in the emergency department is required to reduce overcrowding by sorting, prioritizing, and allocating scarce resources to the medical needs of patients for proper treatment distribution based on the severity of their condition at the time of presentation [5]. As a result, every patient in the emergency department is subjected to a triage system rather than being seen on a first-come, first-served basis. Triage is a crucial system in the emergency department that entails classifying patients based on their priorities to shorten waiting times and enhance patient care [6].

Globally, the emergency department uses different triage systems to evaluate the seriousness and urgency of incoming patients’ conditions and choose the appropriate treatment course. The Manchester Triage System (MTS), the Australian Triage Scale (ATS), the Canadian Triage and Acuity Scale (CTAS), Korean Triage and Acuity Scale (KTAS), and the Emergency Severity Index (ESI) are all examples of triage instruments [6,7,8]. In Africa, many developing countries needed a formal triage system to categorize incoming patients in their emergency departments. Thus many countries have adopted a specific system to enhance the standard of patient care. The Cape Triage Group, known as South African Triage Group (SATG) today, is a designated triage body focusing on improving equity and quality in the emergency department and developed and validated the South African Triage Scale (SATS) [9]. The SATS aims to reduce waiting times for patients with life-threatening conditions and facilitate streaming less urgent patients. Notably, with SATS, patients are classified into red, orange, yellow, green, or blue based on the triage score [10,11]. Those color-coded red are taken to the resuscitation room for immediate management. For instance, (1) orange coded should be treated in less than 10 min for very urgent management, (2) yellow coded should be treated in less than an hour for urgent management, (3) green coded should be treated in less than 4 h in designated area for non-urgent cases, and (4) blue coded are dead and should be certified in less than 2 h. Significantly, patients who have waited longer in the emergency department than expected should be re-triaged as per the triage category.

Multiple African countries have adopted the SATS to fit their setting-specific needs. South Africa, for example, developed and validated the SATS as a triage tool in resource-limited settings to effectively sort and categorize patients in the emergency department [6,12,13]. Subsequently, in 2007, all South African provincial health departments adopted the SATS as a nationwide triage tool [6,14]. Notably, this SATS has since been adopted by numerous emergency departments as it is user-friendly and more suitable for developing countries. This follows the successful use of the SATS in Southern Africa and other African countries, such as Ghana, Rwanda, Ethiopia, and Botswana, which have adopted the system [15,16,17]. In Uganda, Mulindwa and Blitz’s study recommends adapting the SATS in all hospitals in the country as that could improve the emergency care of patients [18]. Subsequently, in Kenya, Wangara et al. study findings demonstrated that the SATS could be effectively implemented in a tertiary public hospital [19]. Despite the benefits of the triage system in the emergency department, there are criticisms against the triage process. These criticisms include that triage is more suitable for directing treatment decisions and forecasting death in groups of patients following significant accidents than for individual patients in an emergency department [6,20,21,22]. In addition, triaging of patients could be biased towards patients who seek emotional support and whose emotions might deteriorate rapidly, at the expense of trauma patients whose vital signs might remain within normal ranges for limited periods, even after major traumatic injuries. For example, a gap in triaging mental health care users was reported in a study by Önnheim, Johansson, Ivarsson, and Hagström, primarily perpetuated by specialist nursing curricula on managing medical illnesses and mental symptoms were frequently overlooked [23].

Different studies reported patients’ experiences concerning the triage system at various healthcare levels. For instance, in the study conducted by Toloo, Aitken, Crilly, and FitsGerald, in Australia, over 48% of the population is expected to be prioritized over the actual triage category, confirming the disparity between the patient perception of urgency and staff assessment of urgency [24]. A study in Kenya demonstrated adequate triage performance after implementing the South African Triage System (SATS) when individual triage acuity categories were assessed. A variable percentage of correct triage answers for very urgent (72%) versus urgent (61%), routine (83%), and emergency categories (51%) were found [19]. Ibrahim conducted a cross-sectional study at the emergency department of eight public hospitals spread across seven localities in Khartoum State-Republic of Sudan to investigate the triage system and identify the barriers to its implementation [25]. Of the 185 participants, 57% admitted that their hospitals had a triage system, but only 50% thought it was efficient and effective [25].

In the Limpopo Province of South Africa, the emergency department of a selected tertiary hospital adopted the SATS. The primary author, a trauma specialist nurse, has observed with concern that since the implementation of the triage system at the emergency department, patients who are triaged yellow and green always complain about the long waiting time. This concern was observed during the primary author’s postgraduate diploma training in emergency nursing (trauma). Although there is an interconnection of literature describing knowledge, skills, and practices of patient triage among emergency nurses, few studies documented the patient’s perceptions concerning the triage system in the emergency department in South Africa. These studies include the one conducted by Adeniji and Mash on patients’ perceptions of the triage system in a primary healthcare facility in Cape Town, South Africa, and the findings of that study reveal that all of the participants complained of a lack of information and poor understanding of the triage process [10]. The current study was conducted in a selected tertiary hospital which serves as a referral center for all complicated emergency medical conditions for all the districts and regional hospitals of the Limpopo Province. Subsequently, many emergency nurses in the same province of Limpopo were not trained in the triage system. For instance, the study by Phukubye conducted in the emergency department of the Sekhukhune District on knowledge and practice of triage among nurses revealed that 61% of the nurses in the emergency department had a poor practice of the triage model despite the model efficacy of sorting patients [26]. However, the findings only focused on nurses while neglecting the patients as recipients of emergency services. Therefore, this paper aims to establish patients’ perceptions regarding triage practice.

To achieve the above aim, we adapted Benner’s original seven domains to describe the perceptions of patients regarding the triage system in emergency departments [27]. The seven domains of Benner’s theory have been widely used to assess nursing practice worldwide, primarily focusing on a novice nurse becoming an expert [28,29,30]. Despite these studies concerned with promoting patient safety, we notice fewer deliberations regarding using Benner’s theory in testing the knowledge and perceptions of the patients as recipients of quality service delivery, including triage systems provided by novice or expert nurses. Therefore, this study can contribute to the growing literature that examines the patient’s perceptions of using the SATS in the emergency department in the region. The study results could also help emergency department management, healthcare providers, hospital administrators, and policymakers find ways to develop and improve triage practices.

## 2. Materials and Methods

### 2.1. Design

The study used a qualitative explorative research design to explore the patient’s perceptions concerning the triage system [31]. The researchers were able to obtain in-depth information about patient perceptions at selected tertiary hospital emergency departments in Limpopo Province, South Africa. The richness and depth of the description gained from a qualitative approach provided a unique appreciation of the reality of the experience [32].

### 2.2. Setting

The study was carried out at the emergency department of the selected tertiary hospital in the level three category in Limpopo province of South Africa. Twenty-two wards, fourteen specialist outpatient clinics, two resuscitation units, trauma, and medical/surgical, one triage area, and twenty resuscitation units are present at this particular tertiary hospital. This selected tertiary hospital serves as a referral center for all complicated emergency medical conditions for all the districts and regional hospitals of the Limpopo Province. Notably, the selected tertiary hospital is situated in Capricorn District. Limpopo province comprises five health districts, namely, Vhembe, Mopani, Capricorn, Sekhukhune, and Waterberg are the five districts that make up the Limpopo Province. The tertiary hospital was chosen as the study setting because it has a significant daily patient influx in the emergency department.

### 2.3. Population and Sampling

A total of 115 patients who were admitted to the emergency department were considered for the study. Sampling is the process by which the researcher chooses a sample from a population to learn more about a phenomenon that accurately represents the population of interest [33]. A purposive sampling technique was used to select patients who had already undergone triage. Triage priority colors include; red, orange, yellow, green, and blue. However, for this paper, the population consisted of all the triaged patients color-coded green (less than 4 h of waiting) and yellow (waiting for an hour or less) as these patients are often stable patients who stay for some time in the emergency department, a minimum of 60 min. Unlike the red, orange, and blue categories, those who could not communicate were excluded from the study for physiological reasons. For example, the red and orange color codes are critically ill patients fighting for their lives with unstable or critical medical conditions, while the blue-coded patients are certified. Still, only those willing to participate were included in this study.

### 2.4. Data Collection

The primary author, skilled in qualitative research, conducted semi-structured one-on-one interviews in clinical settings while using the domains of Benner’s theory as a guide. The second domain, teaching, was not relevant to the study, so only six of the seven domains were employed. The seven domains are as follows: (1) helping role; (2) teaching; (3) diagnostic and patient monitoring function; (4) effective management of rapidly changing situations; (5) administration and monitoring therapeutic intervention regimen; (6) monitoring and ensuring the quality of health care practice; and (7) organizational work role competencies [27]. The study was conducted on hospital premises, in a consulting room with good ventilation and minimal background noise, and where COVID-19 safety regulations, such as social distancing, sanitizing after each participant, and wearing masks, were already in place. The help of the operational managers in the emergency department was used to assist in the recruitment of the participants who had been prioritized for care. The author built rapport with patients by introducing himself and explaining the study’s aim, the benefits of participating in this study, and that participation in the study was entirely voluntary, including the type of people who would be interviewed and the interviewing process.

The data were collected in one week in August 2020. One central question, “Can you please describe your perceptions with regard to the triage system practice in the emergency department?” was posed. Notably, following the initial response, clarification-seeking questions were posed to help the researcher elicit more details regarding the phenomenon under investigation. Each interview session per participant lasted between 30 and 45 min to establish rapport with the participants, and it is noteworthy to mention that all participants were interviewed once, and follow-up questions were asked when the need arose. All interview sessions were audio-recorded on a digital voice recorder, and field notes were taken. Essentially, the primary author used both English and Northern Sotho (Sepedi) to conduct interviews, as some patients could not express themselves in English but rather in their native language. The interview sessions continued until data saturation, at participant number 14. While guaranteeing their privacy, participants’ consent was requested to audio-record the interviews.

### 2.5. Data Analysis

A narrative qualitative analysis was applied to make sense of data by adopting the seven domains of Benner’s theory [27,34]. All audio-recorded interviews were captured verbatim in a Microsoft-word document. Three primary authors attentively read all of the verbatim transcripts independently. They noted any ideas that came to mind concerning the seven domains of Benner’s theory and the study’s objective. The primary three authors coded the common statements relating to the domains as concepts during the verbatim transcriptions. Each concept materialized throughout the scaling-down process and was sorted into the domains of the theory. Concepts relating to one domain were grouped as patients‘ perceptions regarding the triage system of the emergency departments. The results were then compared with the recorded and transcribed data with the help of the last author, who acted as an independent co-coder to ensure data accuracy and reliability.

### 2.6. Trustworthiness

The trustworthiness in this study was ensured using various strategies, as suggested by Lincoln and Guba [35]. This study confirmed credibility by using prolonged engagement with the patients to fully understand their perceptions concerning the triage system in the emergency department of the selected tertiary hospital. The researcher spent at least 40 to 50 min with the caregivers to build rapport by clearly explaining the study’s purpose and the ethical issues involved, equally by recording all the interview sessions and capturing the field notes as proof of the participants’ data collection. The research methodology’s full explanation helped to assure transferability. By safely storing all field notes and audio recordings, dependability was ensured. To ensure confirmability, the three primary authors submitted the verbatim transcripts to the last three. A meeting was set up to discuss the themes and subthemes, in which consensus was reached [36].

### 2.7. Ethical Consideration

The Ethical clearance for this study was obtained from Turfloop Research Ethics Committee (TREC/258/2020: PG). Additionally, permission to conduct this study at the selected tertiary hospital was sought from the Limpopo Provincial Department of Health and the Operational Management of an emergency department. The study’s purpose and goals were explained to the participants, who were informed that participation was entirely voluntary and that they might leave the study at any time without risk of retaliation. Participants were also allowed to sign a standard Informed Consent Form as proof of their agreement with the researcher. Data were kept confidential behind secured cupboards of the primary author, and the digital copy was password-protected. The primary author ensured that the patients’ identities were kept private and not disclosed to anyone outside the research team. That was accomplished by assigning pseudonyms to participants during data collection. During the data collection process, participants were only identified as Participants 1, 2, or 3.

## 3. Results and Discussion of Findings

The paper presented patients’ perceptions of the triage system at a selected tertiary hospital using Patricia Benner’s theory of clinical wisdom in nursing practice and the SATS. The following themes were used to determine patients’ perceptions concerning the triage system in a selected tertiary hospital in the Limpopo province.

### 3.1. Demographic Profile

As depicted in Table 1 below, we only focused on three variables, gender, age, and triage priority, as widely identified as contributory factors in perceiving triage poorly. A total of 14 green and yellow triaged patients were interviewed until data saturation was reached. In total, 6 male and eight female patients participated in the study, with ages ranging between 18 to 51 years old. Notably, 71% (10) of the patients in this study were triaged green, while 29% (4) were triaged yellow.

### 3.2. Perception of Patients concerning the Triage System in This Emergency Department

This paper was grounded in Patricia Benner’s theory of clinical wisdom in nursing practice by using the seven domains, namely (1) helping role, (2) teaching, (3) diagnostic and patient monitoring function, (4) effective management of rapidly changing situations, (5) administering and monitoring therapeutic intervention regimen, (6) monitoring and ensuring the quality of health care practice, and (7) organizational work role competencies [27]. However, there was no patients‘ perception regarding conceptual domain two—teaching. Hence, we discuss the following six domains.

#### 3.2.1. Domain 1: Helping Role

The domain covers competencies such as establishing a healing relationship, providing comfort measures, and encouraging active patient participation and control in care [27]. The authors explored patients’ perceptions regarding triage as the helping role of emergency department nurses to patients. The study findings revealed mixed perceptions of patients regarding the triage system as a helping role in the emergency department. Some patient participants highlighted how helpful the triage was in the emergency department.

This is evident in the assentation by Participant 3, who alludes that *“she was from the clinic. By the time I got here to the emergency department, the condition was stable, but after some time, the child started bleeding, and I reported it to the nurses. They could attend to the child, clean the wound, and dress it and then I went back to the queue, and they explained to me that is what they can do while you are still waiting for the doctor”.* Equally, Participant 12 in this study was satisfied with the use of the triage system in the emergency department, and according to this patient, *“they must continue with the system because it helps save lives and prevents deaths where it could have been prevented but the nurses who are responsible need to communicate with us regarding the time that we will wait before seeing the doctor”*. On the other hand, patients were not satisfied with the use of the triage system in the emergency department. According to Participant 11, *“the nurse said they use this system to help people, but I disagree with it”*. Furthermore, the discontent with the triage system is evident in Participant 14′s assentation that nurses *“are trying but at least [there should] be someone who comes to assess the condition of those waiting in line because some may be very ill but appear stable physically”*.

#### 3.2.2. Domain 3: Diagnostic and Patient Monitoring Function

This domain is more concerned with the competencies of emergency nurses and doctors in providing ongoing patient assessment and anticipation of outcomes [27]. Emergency nurses can effectively detect, diagnose, and classify life-threatening crises in emergency rooms with proper triage systems. That can be achieved through patient history taking, monitoring vital signs, and assigning patients to the appropriate triage codes.

Considering the above discussion about diagnostic and patient monitoring, other participants in this study demonstrated a good perception by stressing that a triage system is essential in the emergency departments, particularly in offering emergency care to critically ill individuals. Participant 1 articulated that the triage system: *“is critical because they help patients who are severely injured more than us. They will also help us at the end.”* It is also worth noting that Participant 1 perceived the triage system as a lifesaving mechanism, and this is what they stated, *“We do not have a problem because they check the type of patient they are and which type I am. If that patient is too sick, they must attend first, and then we will enter.”* Furthermore, among other issues, participants acknowledged emergency nurses’ communication concerning waiting time and reassurance that all patients will receive the healthcare service. Participant 1 asserted, “*my suggestion is that they did the right thing as they know that the patient who is very sick should be seen first before the second one can be seen. They look at the type of patient and the severity of their injuries. If we can say that if I came first, then one who is very sick should not enter, then I will be unhuman”* Furthermore, Participant 4 said that *“the information provided is that with the triage system, they check the severity of injury or illness each patient is presenting within the emergency department.*

On the other hand, other participants were not happy about the triage diagnostic system as they view themselves as humans who deserve the same treatment as others: “*it’s not important because we all left our homes, we are all humans, some of us unsettle ourselves and wake up in the morning because we know that we have things to do, we are here to receive care, those who came after us if they are seen first it means that our plans will wait while we woke up in the morning [Participant 2]”* In addition Participant 10 stated “W*e are not happy because we left our home early, we woke up in the morning telling ourselves that we will come back early and do our things, they will help us early and we will be able to do our things, or we will be able to go to our working places, or we will go where we want to go so when does who came after us after seen first, we are not happy about it, we are all humans, and we have things to do all of us [Participant 10] ”*

#### 3.2.3. Domain 4: Effective Management of Rapidly Changing Situations

The conceptual domain relates to matching demands with resources and assessing and managing care during crises [27]. If nurses are knowledgeable about triage and it is adequately implemented and functional, they will be able to manage every situation within emergency departments. In addition, emergency nurses will have a system that has been demonstrated to categorize emergencies of all kinds, minimize crowding, and shorten patient wait times even in the face of mass casualties. In contrast to this domain, most of the participants in this study deliberated on how unhappy they are with the current triage system in the emergency department of this selected tertiary hospital.

According to Participant 2: “*I am not happy about it because we all came here, so they must not take people according to the severity of their injuries as long as we all left our homes at the same time; we have to queue and move according to the line”*. According to this participant, she is unhappy about the long waiting time and suggests that patients must be assisted on a first-come, first-served basis. Subsequently, Participant 6 did not feel all right about the waiting time, and this is what she had to say *“since I came in the morning, they assessed vital signs and told me to wait for the doctor, and now it is afternoon I am still waiting”.* The participants also perceived that the triage system should only be used during accidents or severe injuries involving many people. The former statement is attested to by Participant 7 when he articulated that *“in case of accidents cases, like a car accident, I see it as a good thing because they are able to help those who need help fast”.* The findings further denote that patients had a mixed perception regarding the triage system to classify incoming patients based on the seriousness and urgency of their health conditions.

#### 3.2.4. Domain 5: Administering and Monitoring Therapeutic Intervention Regimen

This conceptual domain is concerned with preventing further complications during drug therapy, wound management, and hospitalization [27]. After nurses have allocated a triage color code to the patient, appropriate treatment will be implemented immediately, based on the category of patient. The SATS aims to reduce wait times for patients with life-threatening conditions while streaming less urgent patients. Patients are classified as red, orange, yellow, green, or blue based on their triage score for drug therapy, wound management, and hospitalization; however, the perceptions of patients illustrate that treatment was delayed in this study.

For instance, Participant 4 states, *“the triage system of Mankweng hospital makes you wait for a longer time, and I am not happy about it, and someone might end up dying as we wait for more than 3 h. I am not trying to ruin the hospital’s name, but I am unhappy with their service”.* Subsequently, patients in this study shared that they waited hours for doctors after undergoing triage. This is evident when Participant 9 said, *“I do not feel good about waiting for the doctor after triage, as their treatment and service are bad. I know we are not paying enough, but the service is terrible.”* Equally, Participant 11 said, *“I don’t feel good to have to wait for a doctor after triage, and they should fix it, as in the ward it’s written emergency, meaning that they should get help fast and not have to wait for a doctor.”*

#### 3.2.5. Domain 6: Monitoring and Ensuring the Quality of Healthcare Practice

Maintaining safety, improving health quality continuously, collaborating and consulting with physicians, self-evaluation, and technology management are all crucial in this conceptual domain [27]. A triage system within the emergency departments helps to ensure that patients receive quality emergency care, but this is different based on patients‘ perceptions.

Participant 5 states, *“the triage system used in this emergency department is terrible. There is no need to classify patients as emergency and non-emergency because even bleeding patient queue, so I think they are all the same. Also, some patients are attended fast when it is not an emergency just because they have friends or relatives working in this hospital, so you need to have connections for you to be attended fast”.* These participants further allude that the nurses do not check or communicate with them. According to this participant, *“as a patient, you are the one who should go after them asking questions”.* In addition, Participant 8 also expressed concern about the poor implementation of the triage system in the emergency department. According to this participant, *“the whole triage system is terrible. It should be fixed so that things go smoothly as things are currently a mess.”* Similarly, Participants 9 and 8, also raised concerns over the ineffective triage system in the emergency department. According to them, the triage system is inadequate as people might die. Participant 8 states, *“I see the triage system as necessary, but they need to hurry to attend to sick people.”* Another participant also said, *“the triage system is not a good thing, I came in the morning, and then someone is attended to first while I been waiting, whereas I am feeling pain but just being patient.”*

#### 3.2.6. Domain 7: The Organizational and Work-Role Competencies Domain

The organizational and work-role competencies domain prioritize setting, team building, coordinating, and providing continuity of care within the emergency departments [27]. This theory domain advocates for a triage system because it will facilitate appropriate organization despite the staff shortage and resources available to provide quality care where it is due. Participants in this study deliberated how the triage system within the emergency departments is disorganized, which impacts the quality of service delivery.

For instance, Participant 5 alludes that *“there is not much information given to us about where to go after being triaged. They only collected vital data and did urinalysis”.* Subsequently, Participant 6 also said, *“I did not understand the triage system even a little bit. They just told us that they divide patients according to the severity of illness, I came here early in the morning, but people come and leave me here, so I do not see myself understanding it.”* It also was noted and learned from participants that the triage system is beneficial for the emergency department in order to save lives, although there is a dysfunctional existence. According to Participant 7, *“the importance of the triage system is to identify the patient who needs to be attended first, to save lives.”* Further, that was evident in Participant 8 assertions, as she claimed that *“the triage system is essential to ensure order and avoid confusion.”* Therefore, emergency nurses must work as a team to ensure continuity of care and communication, especially after patients have been triaged.

## 4. Discussion

The following variables, gender, age, and triage priority, are significant contributory factors in perceiving triage poorly [37,38]. Similarly, the current study findings demonstrate that more females, mostly 26–35 years old, were triaged during consultation in the emergency department. These results align with those described in the literature, which show that women seek out healthcare more frequently than males due to their biological makeup, including pregnancy and reproductive health care. Age distribution followed patterns noted in other South African studies, with most patients presenting as young adults aged 19–39 [38]. This may be explained by the burden of trauma, pregnancy-related illness, and HIV affecting this age group. The following sections are discussed according to the listed domain of Patricia Benner‘s theory.

### 4.1. Helping Role

The findings of this study denote that participants had a positive perception of the triage practice in the emergency department, which aligns with the helping role domain from Benners‘ theory. Participants further alluded that the system is highly important as it helps save lives and prevent death. The results of this study are consistent with those of a cross-sectional study conducted in a Saudi tertiary hospital, in which 61% of 334 participants reported understanding why some patients are seen before others, and those who understood why some patients are seen before others were more likely to consider the situation fair [39]. It is noteworthy that triaging every patient entering the emergency room was deemed essential by 280 (73%) participants, and 262 (69.50%) participants believe that triage efficiently identifies sick patients and provides prompt management in a study conducted by Rijal and Adhikari in Nepal [40]. Equally, in the study by Alsulimani (2022), the majority of respondents (80%) understood why some patients were taken to a room before others, despite having waited less, and 85.3% thought this was fair [41]. Though the triage system is perceived as efficient and practiced, there is still a need for emergency department staff to improve communication regarding the waiting time. Literature corroborates this study’s findings that emergency nurses should update patients on when they will be attended to and how the triage system works. For example, the majority of patients who had undergone triage in the emergency department of a primary care facility expressed dissatisfaction with their experience, citing issues such as a lack of information from the triage nurses [9]. Therefore, our study findings highlight the importance of the emergency departments aligning with the Batho-Pele Principle (People First), National Government White Paper, highlighting that all customers are entitled to be given information about the services provided and the expectations to it.

### 4.2. Diagnostic and Patient Monitoring Function

The current study revealed that the participants reported good practice regarding monitoring and diagnosing patients in the emergency department. They further perceive the triage system as critical. More importantly, the participants displayed the spirit of ubuntu” *I don’t want to be unhuman’’*. The current study was conducted in Limpopo Province, South Africa, where the communities are informed by diverse rules, values, and beliefs, such as Ubuntu (Humanity to others). Other participants also perceive the triage diagnostic system as one-sided because they are humans and have other plans for the day. For this reason, their paper recommends training emergency nurses in more effective communication skills for modifying help-seeking behavior. In Saudi Arabia, respondents desired more general health information and information about their visit [41]. These expectations should be met through public health campaigns or brochures distributed in emergency rooms. Nurses’ failure to communicate in the emergency departments may result in serious adverse events and contribute to disparities in care, lowering healthcare quality and jeopardizing patient safety [42].

### 4.3. Effective Management of Rapidly Changing Situations

In this study, the realization came that many patients were unhappy with the time it took before a healthcare provider consulted them in the emergency department. These findings relate to the effective management of rapidly changing conditions domain on Benner‘s theory—the unhappiness of patients with the triage practices of nurses indicates the effective implementation of the triage system and a lack of understanding by patients regarding the system. In the study by Toloo et al., the discrepancy between the patient perception of urgency and staff assessment of urgency was noted, whereby over 48% of the respondents expected to be given higher priority than the actual triage category they were assigned [24]. An examination of the literature suggests various factors are barriers to implementing the triage system, particularly in emergency departments. These factors include a patient deficit of knowledge about the triage system’s use, the importance of triage thereof, lack of communication, and a shortage of clinicians in the emergency department. The staff shortage affects the adequate implementation of the triage system. Patients triaged as orange in the emergency department also waited longer than recommended before the clinician could medically attend to the patient. According to the South African Triage Scale, the patient triaged orange should be seen within 10 min, the yellow within 1 h and the green triaged patient should be attended to within 4 h [11]. This is congruent with our study findings, where there was a discrepancy between the actual triage category and the patient’s expectations. Hence the triage system was labeled as “terribly bad” by Participant 5 in the study. When many seek care in the emergency department, a prolonged waiting period of four to six hours delays the patient’s diagnosis.

### 4.4. Administering and Monitoring Therapeutic Intervention Regimen

The study findings confirm prolonged waiting times that impede the triage system’s effectiveness in the emergency departments, which relates to administering and monitoring therapeutic intervention regimens. The triage system also enhances the delivery of time-critical treatment for patients with life-threatening conditions. Further, it ensures that all people requiring emergency care are appropriately categorized according to their clinical condition [9]. Though triage is crucial in reducing overcrowding and prioritizing patients, the participants in this study perceived it as a system that prolongs patients‘ attendance in the emergency department. Therefore, the emergency department needs to practice patient-centered triage, wherein patients will be involved and informed about the triage process. According to participants of this study, some patients were promised to be attended to within 1 h but spent more than 2 h in the emergency department. The patients also revealed that the triage system of the selected tertiary hospital makes individuals wait for a longer time, and some might end up dying while still waiting in the queue for more than the expected hours. The current study findings are similar to those reported in the existing literature. For example, a qualitative study conducted in Botswana by Phiri, Heyns, and Coetzee revealed that patients were dissatisfied with the prolonged waiting times and felt neglected and emotionally distressed due to a lack of communication during the triage process [43].

The findings of this study suggest that poor implementation of the triage system, lack of communication, and patient education on how triage works hinder the effectiveness in the emergency department and acceptance of the triage system among patients. Prolonged waiting times have an impact on the quality of health care services and contradict the South African government’s Batho-Pele (people first) principles and can negatively affect how patients experience care and treatment, compromising patient safety, risking deterioration of their medical condition and causing anxiety and negative patient experiences in the emergency department [44]. There is a clear realization that patients were not attended to within the recommended triage color code, which delays drug therapy, wound management, and hospitalization. This is significant because, according to the sixth ministerial priority, reducing waiting times is a priority for the South African National Department of Health. Despite the challenges impeding the triage system, Phiri emphasizes that it is a lifesaving initiative, especially for critically ill patients, and improves the quality of emergency department services and patient satisfaction [45].

### 4.5. Monitoring and Ensuring the Quality of Healthcare Practice

The current study findings illustrated monitoring and ensuring the quality of the healthcare practice domain as a challenge. Patients’ perceptions demonstrated that triage practice is not performed effectively, which entails that the tertiary hospital’s selected emergency department must reinforce the triage system’s implementation to ensure that patients are attended to within the recommended triage time. This will improve patient satisfaction concerning the quality of healthcare they receive. Therefore, patients’ perceptions demonstrate that monitoring patients and ensuring standard healthcare service in the emergency departments of the selected tertiary hospital still needs to be achieved. In this study, patients who were color-coded green perceived the triage process as biased and unfair, and they also reported that the triage nurses were rude and unprofessional. Those who were color-coded yellow found the triage nurse helpful and professional. Similarly, in the qualitative study conducted by Adeniji and Mash in 24-h community health centers in South Africa [10]. Moreover, many patients perceived the triage system as unfair or biased as they thought nurses were rearranging patients based on whether they knew them or not since they were unaware of the reasoning behind it and lacked information about what was happening.

### 4.6. The Organizational and Work-Role Competencies

The patient perceptions regarding triage practice demonstrated a need to re-design emergency nurses’ organization and work roles. The study findings have revealed that although different triage systems are used worldwide to assess the severity and urgency of incoming patients’ conditions and assign treatment priorities, some patients lack knowledge of triage systems in the emergency department. Lack of knowledge in this research emerged as a vital factor in perpetuating negative attitudes towards a triage system in the emergency department at the tertiary hospital of Limpopo province. Subsequently, the findings of this study reveal that the patients receiving healthcare in the emergency department in South Africa are as likely as those in other counties to understand and define the triage system correctly, including regarding the system as unfair. In the quantitative study conducted in Saudi Arabia, 37 (11%) of the participants reported understanding the triage system with the correct definition, and 13% (43) of the respondent incorrectly defined the triage system or did not provide an answer [39]. More efforts should be made to promote knowledge of the triage system at the tertiary hospital of the Limpopo province.

## 5. Limitation of the Study

As the study only looked at the green and yellow-coded participants, additional interviews encompassing all the color codes of patients may have provided a different perspective, even if data saturation had been reached at Participant 14. When using these findings in a different context, these concerns should be taken into account.

## 6. Conclusions

The six relevant domains illustrated mixed patients’ perceptions regarding the triage system in the emergency departments. Although most of the participants in this study feel that the triage system is biased and unfair, some patients were satisfied with the triage system in the emergency department of the selected tertiary hospital. Hence, the domain helping role of the triage system was overweighed by the dissatisfaction of the needy patients who waited for an extended period to receive emergency services. We conclude that the triage system at the selected tertiary hospital is not welcomed due to its disorganization and patient-related factors in the emergency departments. The findings of this paper are a point of reference for reinforcing the triage system’s implementation and improved quality service delivery by the emergency department healthcare professionals and the department of health policymakers.

Furthermore, the authors propose that the seven domains of Benner’s theory can serve as a foundation for research and improving triage implementation within emergency departments. This study recommends that the selected tertiary hospital develop more effective systems and strategies to enhance patient-centered triage practice. In addition, the recommended strategies could inform the patients about the triage model and waiting times, such as posters, patient health education, and video or electronic information boards. Ongoing in-service training is recommended for all healthcare professionals, including support staff such as administrators and security personnel, to ensure that they provide accurate information when asked for assistance. Constant communication and re-triaging or reassessment of patients who waited for more than the expected time is vital to reassure them.

## Figures and Tables

**Table 1:** Demographic profile.

Demographic Variables	Population, *n* (%)
Gender	
Female	8 (57%)
Male	6 (43%)
Age	
18–20	3 (21%)
21–25	3 (21%)
26–35	5 (36%)
36–49	2 (14%)
>50	1 (7%)
Triage Priority	
Green	10 (71%)
Yellow	4 (29%)

## Data Availability

Data are not shared due to privacy and ethical restrictions.
